# The relationship between inferior vena cava diameter measured by bedside ultrasonography and central venous pressure value

**DOI:** 10.12669/pjms.302.4375

**Published:** 2014

**Authors:** Serenat Citilcioglu, Ahmet Sebe, Mehmet Oguzhan Ay, Ferhat Icme, Akkan Avci, Muge Gulen, Mustafa Sahan, Salim Satar

**Affiliations:** 1 Serenat Citilcioglu, MD, Emergency Medicine Service, Cukurova Dr. Askim Tufekci State Hospital, Adana, Turkey.; 2 Ahmet Sebe, MD, Department of Emergency Medicine, Cukurova University, School of Medicine, Adana Turkey.; 3 Mehmet Oguzhan Ay, MD, Department of Emergency Medicine, Adana Numune Education and Research Hospital, Adana, Turkey.; 4 Ferhat Icme, MD, Department of Emergency Medicine, Ankara Numune Education and Research Hospital, Ankara, Turkey.; 5 Akkan Avci, MD, Department of Emergency Medicine, Adana Numune Education and Research Hospital, Adana, Turkey.; 6 Muge Gulen, MD, Emergency Medicine Service, Eskisehir Yunus Emre State Hospital, Eskisehir, Turkey.; 7 Mustafa Sahan, MD, Department of Emergency Medicine, Elazig Education and Research Hospital, Elazig, Turkey.; 8 Salim Satar, MD, Department of Emergency Medicine, Adana Numune Education and Research Hospital, Adana, Turkey.

**Keywords:** CVP, Emergency, Ultrasonography, Vena cava inferior diameter

## Abstract

***Objective:*** We aimed to present inferior vena cava (IVC) diameter as a guiding method for detection of relationship between IVC diameter measured noninvasively with the help of ultrasonography (USG) and central venous pressure (CVP) and evaluation of patient's intravascular volume status.

***Methods:*** Patients over the age of 18, to whom a central venous catheter was inserted to their subclavian vein or internal jugular vein were included in our study. IVC diameter measurements were recorded in millimeters following measurement by the same clinician with the help of USG both at the end-inspiratory and end-expiratory phase. CVP measurements were viewed on the monitor by means of piezoelectric transducer and recorded in mmHg. SPSS 18.0 package program was used for statistical analysis of data.

***Results:*** Forty five patients were included in the study. The patients had the diagnosis of malignancy (35.6%), sepsis (13.3%), pneumonia, asthma, chronic obstructive pulmonary disease (11.1%). 11 patients (24.4%) required mechanical ventilation while 34 (75.6%) patients had spontaneous respiration. In patients with spontaneous respiration, a significant relationship was found between IVC diameters measured by ultrasonography at the end of expiratory and inspiratory phases and measured CVP values at the same phases (for expiratory p = 0.002, for inspiratory p= 0.001). There was no statistically significant association between IVC diameters measured by ultrasonography at the end of expiration and inspiration and measured CVP values at the same phases in mechanically ventilated patients.

***Conclusions:*** IVC diameter measured by bedside ultrasonography can be used for determination of the intravascular volume status of the patients with spontaneous respiration.

## INTRODUCTION

Determination of intravascular volume status in patients admitted to the emergency department is very important. Central venous pressure (CVP) measurement is used to determine need for or excess volume. An invasive method, such as central venous catheter placement, is required in order to measure the CVP. Complications such as arrhythmias, cardiac chamber injury, vascular-nerve injury, pneumothorax, hemothorax, local bleeding, hematoma, infection, thrombosis, occlusion, pulmonary embolism and post-phlebitic syndrome may occur with catheter placement.^1^^,^^2^ Instead, to use bedside ultrasonography as a non-invasive method for hemodynamic monitoring may be a useful tool for emergency clinicians.

The aim of this study was to evaluate the relationship between inferior vena cava (IVC) diameter measured by ultrasonography (USG) non-invasively and central venous pressure and provide inferior vena cava diameter as a guiding method to evaluate patient's intravascular volume status.

## METHODS

This study was started after confirmation of Çukurova University Faculty of Medicine Ethics Committee. Patients over the age of 18, to whom a central venous catheter was inserted to their subclavian vein or internal jugular vein, who needed invasive hemodynamic monitoring, and admitted to Department of Emergency Medicine between January 2011 - September 2011, were included in the study. Written, informed consent for participation was obtained before ultrasonographic examination by him / herself if the patient was conscious or by relatives if the patient was unconscious. Patients who had not agreed to participate in the study, who had an intra-abdominal pressure over 12 cm H_2_O, and whom we could not visualize the inferior vena cava due to the large body habitus, excessive intra-abdominal bowel gas, or large amounts of intrathoracic air were excluded.

A data collection form was created in order to gather standard data. Patient’s age, sex, CVP and IVC diameter measurement values at inspiration and expiration, disease diagnosis, arterial blood pressure, heart rate, CVP catheter type, status of the patient's spontaneous or mechanical ventilation, airway pressure, tidal volume, respiratory rate, PEEP (Positive end-expiratory pressure), inspiratory pressure and abdominal pressure values were recorded in data collection form.

Measurements were performed in patients with spontaneous breathing or mechanical ventilation. In mechanically ventilated patients, ventilator mode was set to volume controlled mode. Tidal volume is set to be 8 cc / kg, respiratory rate to 12 / min and FiO_2 _- PaO_2 _values to over 100, respectively. PEEP was adjusted to be 5 cm H_2_O.

**Fig.1:**
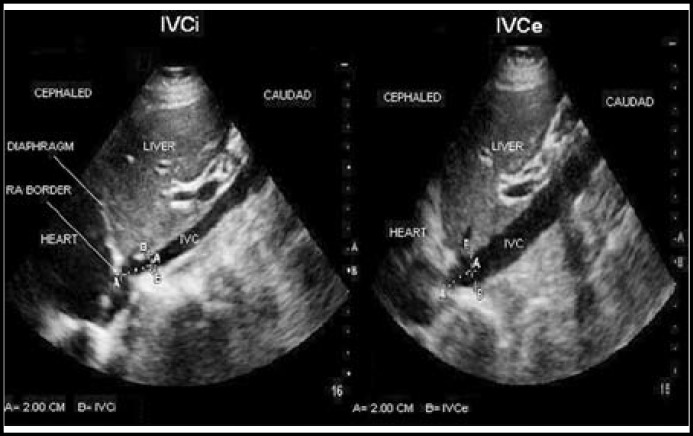
Ultrasonographic determination of inspiratory inferior vena cava (IVCi) and expiratory inferior vena cava (IVCe).

The inferior vena cava diameter measurements were performed in the supine position with Philips HD3 ultrasound device and 3.5 MHz convex probe. Vena cava and aorta were determined with transverse examination by 3.5 MHz probe. Then probe was directed to longitudinal plane over vena cava. Heart was localized with cranial angulation in this plane. Then the point where hepatic veins empties into the inferior vena cava was found by reducing the angulation. The distance between outer limit of the vessel and inner limit of the vessel at the opposite wall was measured 2 to 3 cm from the right atrial border in a long-axis/subxiphoid view in order to standardize and optimize the measurements. Measurements were taken at the end of both inspiratory and expiratory phases and were recorded in millimeters. The method of IVC measurement is shown in Fig.1.

During central venous pressure measurement, level of the right atrium was taken as reference (zero) level. The point at the level of the fourth costal cartilage and on the mid-axillary line was taken as a reference point. Central venous pressure was monitored by piezoelectric transducers on monitor. The clinician who performed measurements with USG was blinded for these CVP measurements. The results of CVP were detected in mmHg.

The bladder was drained by a Foley urinary catheter while the patient in supine position before the measurement of intra-abdominal pressure. Then 50-100 ml of isotonic fluid was injected to the bladder in sterile conditions and the distal portion was clamped. Then, a 18-gauge needle was entered into output of urinary catheter. Needle was connected to a 3-way system and a water manometer. After filled with sterile fluid, the patient side of the manometer was opened. "0" point of the manometer was aligned to patient's pubic symphisis point and the point where the liquid column was read in cm. Thus, the bladder pressure (BP), so the intra-abdominal pressure was determined in cm H2O unit. Patients with an intra-abdominal pressure over 12 cm H_2_O were excluded from the study.

SPSS 18.0 package program was used for statistical analysis of data. Categorical measurements were summarized as number and percentage and numerical measurements were as mean and standard deviation (where necessary median and minimum-maximum). Correlations between continuous measurements were analyzed by Spearman Correlation Coefficient. Statistical significance level for all tests was taken as p < 0.05.

## RESULTS

Forty five patients, to whom a central venous catheter was inserted at Department of Emergency Medicine, were included in the study. Twenty four patients were male (53.3%) and 21 were female (46.7%). Mean age was calculated as 58.3 ± 17.43 years. A subclavian catheter was inserted to 24 patients (53.3%) and a jugular catheter to 21 patients (46.7%). Eleven patients (24.4%) required mechanical ventilation while 34 (75.6%) patients had spontaneous respiration.

The patients had been under follow up most commonly with the diagnosis of malignancy (16 patients, 35.6%), sepsis (6 patients, 13.3%), pneumonia, asthma, COPD (5 patients, 11.1%) (Table-I).

The mean systolic arterial pressure value of the patients’ was 108.4 mmHg and the standard deviation was 32.173. Minimum systolic arterial pressure value was 45 mmHg and maximum systolic arterial pressure value was 203 mmHg. Mean diastolic arterial blood pressure value of all patients was 60.8 mm Hg, the standard deviation was 15.008. The minimum diastolic arterial pressure value was 30 mmHg; maximum systolic arterial pressure value was 90 mmHg. The average heart rate of patients was 98.87 / min, the standard deviation was 26.403. Minimum heart rate was 54 / min, maximum heart rate value was 172 / min.

Patients, who were included in the study, were divided into two groups as patients with spontaneous breathing and mechanically ventilated patients, and IVC diameter values and CVP values at inspiration and expiration were compared separately (Table-II).

In group of patients with spontaneous breathing, a statistically significant relationship was found between average IVC diameter (22.323±8.655 mm) measured by ultrasonography at the end of expiration and average CVP (7.650±4.836 mmHg) at expiration (p = 0.002).

In group of patients with spontaneous breathing, a statistically significant relationship was found between average IVC diameter (15.097±8.047 mm) measured by ultrasonography at the end of inspiration and average CVP (5.410±4.711 mmHg) at inspiration (p = 0.001).

In mechanically ventilated patients group, a statistically significant relationship was not found between average IVC diameter (14.272±3.495 mm) measured by ultrasonography at the end of expiration and average CVP (8.730±5.676 mmHg) at expiration (p = 0.524).

In mechanically ventilated patients group, a statistically significant relationship was not found between average IVC diameter (16.636±4.410 mm) measured by ultrasonography at the end of inspiration and average CVP (10.550±5.610 mmHg) at expiration ((p = 0.192).

## DISCUSSION

In patients admitted to the emergency department, CVP should be monitored in cases of shock, circulatory failure, massive infusion or transfusion requirement, situations with massive bleeding risk, situations where careful fluid resuscitation is a must such as in pediatric patients or patients with cardiac problems.^3^ CVP is a value indicating right atrial pressure or right ventricular filling pressure. In its simplest terms, CVP is an indicator of intravascular fluid status and right heart function. In normal humans, changes in CVP are correlated with changes in left ventricular filling pressure. There are many factors affecting the value of CVP, such as cardiac performance, blood volume, vascular tone, increased intra-abdominal or intrathoracic pressure and vasopressor therapy.^4^ For this measurement, an invasive method such as a central venous catheter placement is required. Central venous catheter placement, an invasive procedure, has a 15% risk for early and late complications.^5^^,^^6^ In addition to these complications, there are some disadvantages such as prolonged hospitalization, increase in health care costs, reduced quality of life. For this reason, the use of a reliable non-invasive method for hemodynamic monitoring is needed.

The inferior vena cava is the biggest vein of venous system with low-pressure. The expansion of the vein reflects venous pressure changes to a certain extent. This change also reflects the excess of the intravascular volume. For this reason, the inferior vena cava diameter may be an important diagnostic tool in evaluation of hypovolemia and hypervolaemia.^7^ Motions and size of IVC changes with respiration and total body fluid.^8^^,^^9^ Intrapleural pressure becomes negative during inspiration and causes an increase in venous return to the right side of the heart. A decrase happens in intraluminal pressure. Natori et al demonstrated that lumen of inferior vena cava begins narrowing at the beginning of the inspiratory, reaches the narrowest diameter at the end of inspiration, expands during expiration, the respiratory changes on inferior vena cava diameter reverse during Valsalva maneuver and positive pressure ventilation due to increase in intrathoracic pressure and inferior vena cava diameter measurement by ultrasonography is a valuable method for predicting central venous pressure.^10^

**Table-I:** The number and the percentage distribution of the patient's diagnosis

*Diagnosis*	*Number (n=)*	*Percentage (%)*
Malignancy	16	35.60
Sepsis	6	13.30
Pneumonia, COPD	5	11.10
Acute renal failure	4	8.90
Chronic liver disease	3	6.68
Meningitis	2	4.44
Gastrointestinal bleeding	2	4.44
SLE	1	2.22
Pulmonary embolism	1	2.22
Ulcerative colitis	1	2.22
The operation of renal transplantation	1	2.22
Diabetic ketoacidosis	1	2.22
Autoimmune hemolytic anemia	1	2.22
Hypercalcemia	1	2.22

n= number of patients; COPD= Chronic obstructive pulmonary disease;

SLE= Systemical Lupus Erythematosus.

**Table-II:** Comparison of IVC and CVP pressures

	*Number (n=)*	*Mean value*	*Standard deviation*		*p value*
*In patients with spontaneous breathing*					
Expiratory diameter of IVC (mm)	34	22.323	8.655		0.002
Expiratory pressure of CVP (mmHg)	34	7.650	4.836		
Inspiratorydiameter of IVC (mm)	34	15.097	8.047		0.001
Inspiratory pressure of CVP (mmHg)	34	5.410	4.711		
*In patients with mechanical ventilation*					
Expiratory diameter of IVC (mm)	11	14.272	3.495	0.524	
Expiratory pressure of CVP (mmHg)	11	8.730	5.676		
Inspiratorydiameter of IVC (mm)	11	16.636	4.410	0.192	
Inspiratory pressure of CVP (mmHg)	11	10.550	5.610		

IVC= inferior vena cava; CVP= central venous pressure.

In our study, in patients with spontaneous respiration, a significant relationship between IVC diameters measured by ultrasonography at the end of expiratory and inspiratory phases and measured CVP values at the same phases. However, no such relationship was seen in mechanically ventilated patients. In patients with spontaneous breathing, IVC diameter measurement by non-invasive bedside ultrasonography method may provide an idea about CVP pressure, intravascular fluid deficit or excess of patients. In other studies, IVC diameter of patients with heart failure, dependent on hemodialysis, traumatic hemorrhagic shock, sepsis and dehydration were found to correlate with CVP pressure. IVC diameter measurement is a valuable method for clinician to guide the treatment and follow-up in cases of septic shock, decompensated heart failure, acute or chronic renal failure, insufficient fluid intake, other causes of hypotension (hypotension due to vasodilatation such as right heart failure, hypovolemia, pulmonary embolism, cardiac tamponade, tension pneumothorax, myocardial infarction, anaphylaxis, spinal shock, antihypertensive poisoning).

The difference of our study from the others in the literature is the study group. Our study group included the patients admitted to the emergency department with emergency conditions whereas the others were including different study groups such as hemodialysis patients, patients with hemorrhagic shock due to trauma, patients during cardiac surgery with ongoing conditions.

IVC was first shown by Weil as dilated in patients with right heart failure.^8^ In a study conducted by Tetsuka and colleagues, IVC diameter, circulating blood volume and body weight were found to be decreased by ultrafiltration in patients undergoing hemodialysis and especially a correlation was found between end-expiratory diameter of IVC and circulating blood volume.^11^ Kusaba et al, in their study including 28 chronic hemodialysis patients, observed gradual reduction in expiratory inferior vena cava diameter during dialysis compatible with the the amount of fluid taken and observed an increase after blood reinfusion following hemodialysis, and they have proved that the changes in IVC expiratory diameter are more significant than changes in IVC inspiratory diameter.^12^

In a study conducted by Yanagawa et al; 30 patients with hemorrhagic shock due to trauma were compared in terms of IVC diameters measured by ultrasonography at admission and after fluid resuscitation, inadequate expansion in IVC diameter was found to be a possible indicator of inadequate circulating blood volume even if blood pressure was normal.^13^

Lyon et al have found about 5 mm decrease in inspiratory and expiratory diameter of IVC following a 450 ml of blood donation by donors in a study on 31 voluntary people for detection of relationship between circulating blood volume decrease and a reduction in IVC diameter. They reported that assessment of IVC diameter by USG in patients with intravascular volume depletion due to trauma or other reasons may be beneficial.^8^

Lorsomradee et al performed a study on 70 patients in order to determine the correlation between inferior vena cava diameter and central venous pressure during cardiac surgery. They found a correlation between CVP and IVC diameter in patients with CVP equal to or smaller than 11 mmHg.^14^ Marcelino et al also detected a correlation between CVP and caval index^15^ obtained by measurement of IVC diameter of 477 patients followed in surgical or medical intensive care units.^16^ Minutiello and colleagues compared CVP and caval index of 65 patients in their study and reported that caval index is in an inverse relationship with CVP value, and CVP is normal if caval index is ≥ 20% and is increased if caval index is < 20 %.^17^

In our study, in 11 mechanically ventilated patients, there was no statistically significant relationship between IVC diameters measured by ultrasonography at the end of expiratory and inspiratory phases and measured CVP values at the same phases. Arthur et al found a statistically significant correlation between IVC and CVP in a 95 patient-included study in order to determine the relationship between central venous pressure and inferior vena cava diameter measured by transesophageal echocardiography in mechanically ventilated patients under general anesthesia.^18^ In a study conducted by Lorsomradee and colleagues in 2007, after setting the PEEP value to 5 and 10 cm H_2_O during cardiac surgery, they found an increase in CVP and inferior vena cava diameter when PEEP value increased to 10 cm H_2_O from 5 cm H_2_O, but they could not find a correlation between CVP and the inferior vena cava diameter during PEEP ventilation.^14^ We think that the small number of mechanically ventilated patients in our study, no disconnection of patients from mechanical ventilator during IVC measurement, differences in modes and settings of mechanical ventilator seem to be effective in not finding a significant correlation between IVC diameter and CVP in mechanically ventilated patients. As possible changes in pleural pressure during mechanical ventilation will affect central venous pressure, patients with this condition must be separated from the ventilator during the measurement.^19^

## CONCLUSION

It was found that in patients with spontaneous respiration there is a significant relationship between IVC diameters measured by bedside ultrasonography at the end of expiratory and inspiratory phases and measured CVP values at the same phases and IVC diameter measurement can be used for the determination of intravascular volume status. It was found that this method can not be used in mechanically ventilated patients. This study will be useful regarding contribution to clinical experiences and appropriate treatment by emergency physicians with providing intravascular volume status of patients non-invasively. With more detailed and comprehensive studies, this method can be used as a routine.

## Authors’ Contributions:

SC and AS conceived the idea and designed the study. AS and MOA were involved in the making of questionnaire and data collection. FI and AA analyzed the data. Interpretation of data was performed by all authors. MG and MS are secondary intellectual contributors. MOA and SS prepared the initial draft of the manuscript followed by critical revision of manuscript for important intellectual content by all the authors. Consensus of all authors was reached in finalization of draft for publication.

## Notes

***Statement of conflicts of interest: ***The authors stated no conflict of interest.
